# Safety and efficacy of combining transcervical fibroid ablation with operative hysteroscopy

**DOI:** 10.20452/wiitm.2025.17974

**Published:** 2025-08-05

**Authors:** Elvin Piriyev, Angelika Dieter, Sven Schiermeier, Stefan Renner, Thomas Römer

**Affiliations:** Department of Gynecology and Obstetrics, University Witten‑Herdecke, Witten, Germany; Department of Obstetrics and Gynecology, Academic Hospital Cologne Weyertal University of Cologne Cologne Germany; Department of Obstetrics and Gynecology, Sindelfingen‑Boeblingen Clinic, Boeblingen, Germany; Department of Gynecology and Obstetrics University of Cologne Cologne Germany

**Keywords:** fibroid, hypermenorrhea, hysteroscopic resection, operative hysteroscopy, transcervical fibroid ablation

## Abstract

**INTRODUCTION:**

Minimally-invasive, organ-preserving techniques for uterine fibroid management have gained popularity. This study assesses the safety and effectiveness of combining transcervical fibroid ablation (TFA) with operative hysteroscopy (HSC).

**AIM:**

We aimed to evaluate whether a combined approach that involves performing TFA and operative HSC during a single session increased the intra- and postoperative risks.

**MATERIALS AND METHODS:**

A total of 200 women were included in this retrospective study. They were divided into 2 groups: 100 underwent combined TFA and operative HSC (group 1), and 100 underwent HSC alone (group 2). Surgical procedures included fibroid / polyp resection, endometrial ablation, and septum dissection. Outcomes assessed comprised intra- and postoperative complications and symptom improvement.

**RESULT:**

Complication rates were low and comparable in both groups. Among the patients in group 1 with available follow-up (n = 60), 83.3% reported improvement in bleeding symptoms, particularly those treated with TFA combined with myomectomy (84%). The combined approach enabled treatment of a broader range of fibroid types (International Federation of Gynecology and Obstetrics score 0–6) and intrauterine pathologies in a single session. No severe or life-threatening complications were observed. The risk of bleeding during fibroid resection was reduced when the resection was preceded by TFA (*P* = 0.001).

**CONCLUSION:**

Combining TFA and HSC appears to be a safe, effective, and versatile approach for treating uterine fibroids and intrauterine pathologies. It offers procedural efficiency, broadens treatment eligibility, and may reduce the need for reintervention.

## INTRODUCTION

Uterine fibroids (leiomyomas) are the most common benign tumors of the female reproductive tract, affecting up to 70% of women by the age of 50 years.^1^ While often asymptomatic, many women experience abnormal uterine bleeding, especially menorrhagia, which can lead to anemia and fatigue.^2^ Fibroids may also cause pelvic pain, pressure, and urinary or bowel symptoms due to compression of adjacent organs.^1^ In addition, submucosal and intramural fibroids that distort the uterine cavity are associated with infertility, miscarriage, and obstetric complications, such as preterm labor.^1^;^2^

Given the variability in symptom severity based on fibroid size and location, individualized treatment strategies are essential. Management options range from medical therapy to minimally-invasive and uterus-preserving approaches, including ultrasound-guided transcervical fibroid ablation (TFA) and hysteroscopic resection.^3^ The Sonata system (Gynesonics, Redwood City, California, United States) integrates intrauterine sonography and radiofrequency energy for precise, image-guided fibroid ablation, with no incisions and treatment duration of 1–7 minutes.^3^;^4^ Approved by the Food and Drug Administration and CE-marked, the system has no contraindications regarding future fertility and has shown good safety, significant symptom improvement, and a low reintervention rate of 8.2% over 36 months.^3^;^4^;^5^;^6^;^7^ Although not officially approved for adenomyosis, positive outcomes in patients with focal adenomyosis have also been reported.^8^;^9^;^10^

Managing large uterine fibroids presents a clinical challenge, particularly when patients desire uterine preservation and minimal invasiveness. TFA has emerged as an effective treatment option even for fibroids measuring 5 cm or larger. In recent studies, TFA demonstrated significant clinical efficacy in women with abnormal uterine bleeding associated with large fibroids.^11^;^12^ Our previous study reported substantial reductions in symptom severity following the procedure, with most patients experiencing marked improvement in bleeding patterns and quality of life (QoL). Importantly, TFA offers a minimally-invasive, incisionless approach that reduces the risks and shortens the recovery time, as compared with more invasive surgical interventions, such as myomectomy or hysterectomy. This is particularly relevant for large fibroids, which often require extensive surgical intervention under traditional treatment paradigms. The real-time ultrasound guidance and precise targeting of fibroid tissue during TFA contribute to effective volume reduction without compromising healthy uterine tissue. Additionally, the safety profile of TFA in the treatment of large fibroids has been favorable, with low complication rates and a quick return to normal activities. These findings support the growing role of TFA as a viable alternative for managing large fibroids in women seeking symptom relief while avoiding major surgery.^11^;^12^

Treatment of uterine fibroids in high-risk patients, such as those with bleeding disorders, poses significant challenges due to increased perioperative risks. Traditional surgical treatment options may lead to excessive bleeding and other complications, making minimally-invasive approaches particularly valuable. We investigated the use of TFA in patients with known bleeding disorders and demonstrated that this technique offered a safe and effective alternative to conventional surgery.^13^ The study highlighted that TFA, performed without the need for incisions, minimizes intraoperative blood loss—a critical consideration for patients at an elevated hemorrhagic risk. Under ultrasound guidance, TFA allows for precise energy delivery to fibroid tissue, reducing procedural trauma and maintaining hemostasis throughout treatment. Clinical outcomes in this high-risk cohort were encouraging, with significant symptom relief and no major perioperative complications reported. Furthermore, the ability to perform TFA in an outpatient setting under conscious sedation reduces the risks associated with general anesthesia, which can be problematic for patients with complex medical histories. These results underscore the value of TFA as a uterus-sparing, low-risk treatment strategy for managing symptomatic fibroids in patients who are poor candidates for surgery due to bleeding disorders or other comorbidities.^13^

Operative hysteroscopy (HSC) has become an essential tool in modern gynecologic practice and is now widely regarded as the gold standard for the diagnosis and treatment of most benign intrauterine pathologies. This minimally-invasive technique allows direct visualization of the uterine cavity, enabling precise and targeted interventions, while minimizing trauma to surrounding tissues. Among its most common indications are the resection of submucosal fibroids (hysteroscopic myomectomy), removal of endometrial polyps, endometrial ablation for the management of abnormal uterine bleeding, and hysteroscopic metroplasty for the correction of congenital uterine anomalies, such as septate uterus.^14^;^15^;^16^;^17^ The evolution of hysteroscopic instruments and techniques, including the development of smaller-diameter hysteroscopes and bipolar electrosurgical systems, has further improved the safety and efficacy of operative HSC, allowing for many procedures to be performed in outpatient settings without general anesthesia.^18^;^19^ Additionally, hysteroscopic approaches offer faster recovery times, reduced postoperative pain, and lower complication rates than more invasive surgical alternatives, making them an attractive option for both patients and health care providers. As a result, operative HSC continues to play a pivotal role in the management of intrauterine pathologies, contributing significantly to fertility preservation and the overall improvement of women’s reproductive health outcomes.^14^;^15^;^16^;^17^;^18^;^19^

Both HSC and TFA are associated with low complication rates.^3^;^20^ To the best of our knowledge, our previous report—which included a small case series—is the only publication to date describing the combined use of TFA and HSC.^21^

## AIM

The primary objective of the present study was to evaluate, in a larger case series, whether the intraoperative risk increased when TFA and HSC were performed during a single session. The secondary objective was to assess whether a combined approach also elevated postoperative risks.

## MATERIALS AND METHODS

**Figure 1:**
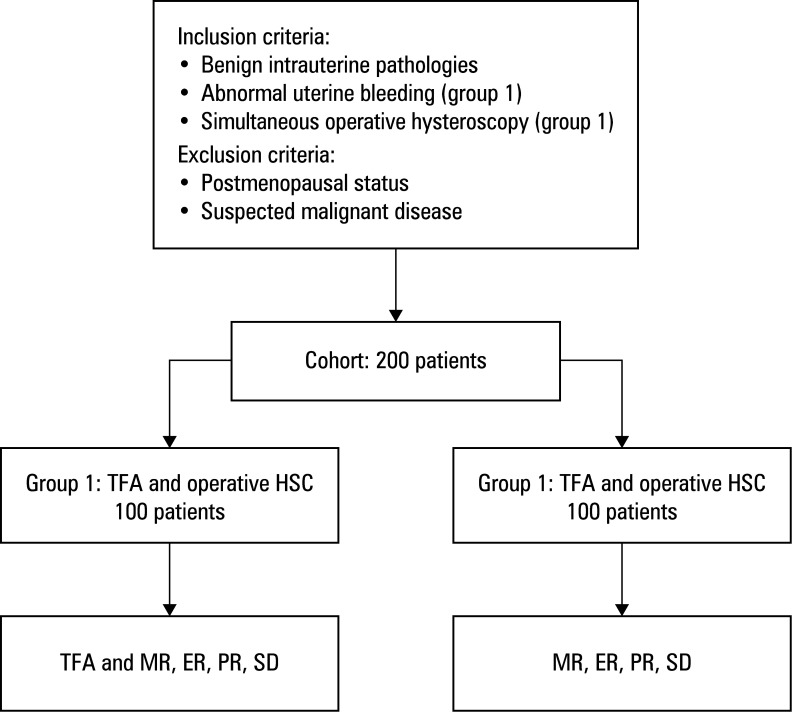
Flowchart of patient selection and grouping

Abbreviations: ER, endometrial resection; HSC, hysteroscopy; MR, myoma resection; PR, polyp resection; SD, septum dissection; TFA, transcervical fibroid ablation

This study included a total of 200 patients who underwent surgical management of benign intrauterine pathologies. The study population was divided into 2 groups based on the type of surgical intervention performed Figure 1, where group 1 was the experimental group and group 2 were controls. Group 1 comprised patients who underwent a combined treatment approach utilizing TFA performed with the Sonata system in conjunction with operative HSC. The procedures were performed at 2 specialized centers in Germany: Academic Hospital Cologne Weyertal and Hospital Böblingen. Group 2 included patients who underwent operative HSC alone. All types of operative hysteroscopic procedures were represented in both groups, including hysteroscopic myomectomy for fibroid resection, polypectomy for endometrial polyps, endometrial ablation for abnormal uterine bleeding, and hysteroscopic metroplasty for uterine septum dissection. The control patients were retrospectively selected from the hospital database using simple random sampling. Inclusion criteria for both groups were: premenopausal status, presence of symptomatic benign intrauterine pathology (eg, fibroids, polyps, or uterine septum), and eligibility for operative HSC. Exclusion criteria were postmenopausal status to ensure a comparable premenopausal cohort and suspected malignant disease. In addition, for group 1, presence of at least 1 fibroid classified as type 2–6 according to the International Federation of Gynecology and Obstetrics (FIGO; accessible for transcervical ablation) was required. Data for group 1 were collected between January 2020 and October 2024. For group 2, data were randomly collected from records of patients treated in 2022.

The final sample size was determined retrospectively and included the first 100 consecutive women who underwent combined treatment with TFA and operative HSC at our institutions. To allow a comparative analysis, a control group of 100 patients who underwent operative HSC alone was selected using a 1:1 matching ratio. A post-hoc power analysis, based on the observed difference in intraoperative bleeding rates between the groups, showed an achieved statistical power of 87.2%, indicating adequate power to detect the observed effect.

The diagnosis of uterine fibroids was established preoperatively through transvaginal sonography, allowing for accurate assessment of fibroid size, location, and suitability for transcervical management. During surgical intervention in group 1, the TFA system was successfully positioned without technical difficulties. Following fixation of the fibroid using the central spike introducer, ablation and safety zones were carefully defined under real-time imaging guidance. The electrodes were then deployed, and, after reconfirming the safety zone, ablation was initiated upon reaching the target temperature of 105 °C. The energy delivery ensured precise thermal coagulation of fibroid tissue while preserving the surrounding healthy myometrium.

Operative HSC was performed according to standard protocols using either bipolar or monopolar resectoscopic systems, depending on surgeon preference and equipment availability. Both intra- and postoperative complications were meticulously recorded, along with postoperative morbidity and clinical outcomes. Significant intraoperative bleeding was defined as bleeding necessitating the use of a Foley catheter for tamponade.^22^ Hemodynamic instability was documented based on the real-time assessment and feedback of an anesthesiologist, including parameters such as blood pressure drops and heart rate changes. In group 1, special attention was given to the assessment of symptom improvement, particularly the reduction in abnormal uterine bleeding. Follow-up was conducted through routine postoperative outpatient visits or, when necessary, via structured telephone interviews to assess patient satisfaction, symptom resolution, and any delayed complications. Improvement in bleeding symptoms was assessed based on subjective perception of the patients who were asked to report whether they experienced a reduction in menstrual bleeding volume, duration, or severity, as compared with their preoperative baseline.

### Data collection

The following data were extracted from hospital records and operative reports: patient age at the time of surgery, body mass index (BMI) calculated preoperatively, fibroid size based on preoperative transvaginal ultrasound, number of fibroids recorded pre- and intraoperatively, FIGO fibroid classification assessed on ultrasound and confirmed during surgery, abnormal uterine bleeding (AUB) type (hypermenorrhea, menorrhagia, spotting) determined based on patient-reported symptoms and clinical evaluation, polyp size and septum length based on preoperative transvaginal ultrasound and intraoperative measurements using hysteroscopic visualization, and procedure type classified as myomectomy, polypectomy, endometrial resection, or septum dissection.

### Statistical analysis

Statistical analysis was performed using IBM Statistics software for Windows, version 25.0 (IBM Corp., Armonk, New York, United States). Categorical variables were analyzed using the 2-tailed Fisher exact test, with descriptive statistics and a confidence interval of the mean (SD), as well as the *t *test to compare 2 means. The data are given as mean (SD). Normally distributed variables are presented as mean (SD), while skewed variables are reported as median (interquartile range [IQR]). This approach was applied specifically to time-related variables, including follow-up duration and radiofrequency ablation time. A *P* value below 0.05 was deemed significant.

### Ethical approval

This retrospective study was conducted in accordance with §15 of the professional code of the North Rhine Medical Association.

## RESULTS

A total of 200 patients were enrolled, with 100 patients undergoing combined treatment with TFA and operative HSC (group 1), and 100 patients undergoing operative HSC alone (group 2). Patient characteristics are outlined in TABLE 1.

**TABLE 1:** Comparison of patient characteristics between group 1 (transcervical fibroid ablation and hysteroscopy) and group 2 (hysteroscopy alone)

Parameter		Group 1 (n = 100)	Group 2 (n = 100)	*P* value
Age, y, mean (SD)		42.06 (6.52)	41.8 (8.26)	0.81
BMI, kg/m², mean (SD)		26.17 (5.85)	26.9 (6.85)	0.42
AUB type, %	Hypermenorrhea	89	92	–
	Menorrhagia	8	–	
	Spotting	3	–	
History of habitual abortions, n		N/A	16	-
Fibroids per patient, n		1 (n = 35)	1 (n = 51)	-
		2–4 (n = 51)	2 (n = 2)	
		>4 (n = 14)	3 (n = 1)	
Fibroid classification 0–2	0–2	92 fibroids	58 fibroids	–
(FIGO types)	3–4	49 fibroids	–	
	5–6	38 fibroids	–	
	2–5	42 fibroids	–	
	N/A	51 fibroids	–	
Fibroid size, cm, mean (SD)		2.99 (1.29)	2.21 (1.08)	<⁠0.001
Ablation time, min, median (IQR)		8.57 (3.4–13.73)	–	–
Follow-up, mo, median (IQR)		7.09 (4.13–10.05)	–	–
Polyp size, cm, mean (SD)		N/A	1.75 (0.72)	–
Septum length, cm, mean (SD)		2.38 (0.48)	3.18 (1.66)^a^	0.36

**a **Includes 2 cases of septus completus

Abbreviations: AUB, abnormal uterine bleeding; BMI, body mass index; FIGO, International Federation of Gynecology and Obstetrics; IQR, interquartile range; N/A, not available; others, see Figure 1

In group 1, mean (SD) age was 42.06 (6.52) years, and mean (SD) BMI was 26.17 (5.85) kg/m^2^. AUB was the primary indication for intervention in all cases, with hypermenorrhea reported in a majority (89%) of the patients. Notably, most patients presented with multiple fibroids, and mean (SD) fibroid size was 2.99 (1.29) cm. Fibroids were classified according to the FIGO system, with a broad distribution across types, indicating a diverse range of fibroid locations and sizes treated. Median (IQR) radiofrequency ablation time was 8.57 (3.4–13.73) minutes, reflecting variability based on the number and size of fibroids treated. Median (IQR) follow-up was 7.09 (4.13–10.05) months, ranging from 3 to 24 months, allowing for the assessment of both short- and midterm clinical outcomes.

In group 2, mean (SD) patient age was 41.8 (8.26) years, and mean (SD) BMI was 26.9 (6.85) kg/m^2^, with no differences in comparison with group 1. AUB was also the most common indication for intervention (n = 92), and a notable proportion of patients (16%) had a history of habitual abortion, which may indicate a higher prevalence of structural uterine abnormalities in this cohort. Mean (SD) fibroid size was smaller than in group 1 (2.21 [1.09] vs 2.99 [1.29] cm; *P *<⁠0.001), and additional procedures, such as polyp resection and septum dissection, were more frequently performed in this group. Mean (SD) polyp size was 1.75 (0.72) cm, and mean (SD) septum length in the cases requiring metroplasty was 3.18 (1.66) cm, including 2 cases with complete uterine septa.

Regarding procedural details, the most common intervention in group 1 was the combination of TFA with myomectomy (n = 73), followed by TFA with endometrial resection (n = 11; TABLE 2). In contrast, the patients included in group 2 primarily underwent myomectomy (n = 43) and endometrial resection (n = 36), reflecting standard hysteroscopic management without adjunctive energy-based ablation. The diversity of procedures in both groups highlights the individualized approach adopted based on patient pathology and clinical presentation.

**TABLE 2:** Intergroup comparison of treatment modalities

Procedures in group 1, n		Procedures in group 2, n	
Total	100	Total	100
TFA + MR	73	MR	34
TFA + ER	11	ER	36
TFA + MR + ER	4	MR + ER	6
TFA + PR	5	PR	7
TFA + MR + PR	3	MR + PR	3
TFA + SD	4	SD	14

Abbreviations: see Figure 1

The overall complication rates were low and comparable between the groups (group 1, 8%; group 2, 11%; *P* = 0.63; TABLE 3). Intraoperative complications, such as uterine perforation and endometritis, occurred at low rates in both groups. Notably, intraoperative bleeding was considerably more frequent during myomectomy procedures in group 2 (14%) than in group 1, where no cases of bleeding during myomectomy were reported (*P* = 0.001). Complications were recorded intraoperatively and during the early postoperative period (2–4 weeks after the procedure). Fibroid expulsion events, however, occurred within 1–6 months post-treatment.

**TABLE 3:** Intergroup comparison of complication rates in combined transcervical fibroid ablation and operative hysteroscopy (group 1) vs operative hysteroscopy alone (group 2)

Complication	Group 1 (n = 100)	Group 2 (n = 100)	*P* value
Endometritis	2 (2)	1(1)	>0.99
Perforation	2 (2)	1 (1)	>0.99
Total intraoperative bleeding	1 (1)	6 (6)	0.12
Intraoperative bleeding after ER^a^	1 (6.7)		0.26
Intraoperative bleeding after MR^b^		6 (14)	0.001
Intraoperative hemodynamic instability	1 (1)	2 (2)	>0.99
Postoperative fibroid expulsion^b^	2 (2)	1 (2.3)	>0.99
Total	8 (8)	11 (11)	0.63

Data are presented and numbers (percentages).

**a **Calculated for the patients undergoing TFA + ER or TFA + MR + ER (n = 15)

**b **In group 2, the values were calculated for the patients undergoing MR, alone or in combination with ER/PR (n = 43).

Abbreviations: see Figure 1

Many patients were referred from various regions across Germany, and due to long travel distances, they did not return for in-person follow-up. Some of these patients could not be reached by telephone despite repeated attempts. Consequently, follow-up data were available for 60% of the patients in group 1. Among those who were followed, 83.3% (n = 50) reported considerable improvement in bleeding symptoms. The highest response rate was observed in the patients subjected to TFA combined with myomectomy (84%), indicating the potential synergistic benefit of combining mechanical removal with thermal ablation of fibroid tissue. Additionally, the combination of TFA with multiple interventions (eg, myomectomy and polyp resection) yielded excellent outcomes, with all patients in these subgroups reporting symptom improvement. Vaginal fibroid expulsion was recorded in 2 cases during follow-up, a known event following thermal ablation procedures (TABLE 4). Follow-up information on symptom improvement was not available or not systematically collected for group 2.

**TABLE 4:** Follow-up and outcomes in the patients undergoing combined transcervical fibroid ablation and operative hysteroscopy (group 1)

Procedure	Patients treated, n	Patients with follow-up data, n	Improvement of bleeding disorders, n (%)
TFA + MR	73	44	37 (84)
TFA + ER	11	4	2 (50)
TFA + MR + ER	4	3	3 (100)
TFA + PR	5	4	3 (75)
TFA + MR + PR	3	3	3 (100)
TFA + SD	4	2	2 (100)
Total	100	60	50 (83.3)

Abbreviations: see Figure 1

## DISCUSSION

This study evaluated the safety and clinical outcomes of combining TFA with operative HSC in women presenting primarily with AUB due to fibroids and other intrauterine pathologies. The results of this retrospective cohort of 200 patients demonstrate that the combined approach is both safe and effective, with complication rates comparable to those of operative HSC alone and a high rate of symptom improvement during follow-up.

Minimally-invasive, uterus-sparing therapies for fibroids have gained increasing popularity as alternatives to traditional surgical options, particularly in women seeking fertility preservation or those who wish to avoid hysterectomy.^23^ TFA offers a unique, incisionless solution that integrates intrauterine ultrasound guidance with targeted volumetric ablation. It has been shown to markedly reduce menstrual bleeding, improve QoL, and facilitate rapid recovery.^3^;^4^;^6^;^7^ Prior studies have shown that sonography-guided TFA produces substantial and durable improvements in fibroid-related symptoms and QoL. For example, the SONATA (Sonography-Guided Transcervical Ablation of Uterine Fibroids) trial^24^ reported a mean symptom severity score reduction of approximately 33 points (from approx. 55 to approx. 22 points) and a health-related QoL increase of 43 points (from 40 to 83 points) at 3 years post-procedure (both *P *<⁠0.001). These gains exceeded minimally-important clinical differences and were accompanied by high patient satisfaction (94% at 3 years postoperatively).^24^ Operative HSC remains the gold standard for the treatment of numerous intrauterine conditions, including submucosal fibroids, polyps, and uterine septa.^14^;^15^;^16^ A combined approach that involves performing operative HSC and TFA during a single session additionally allowed for direct treatment of intrauterine pathologies, potentially enhancing overall symptom relief and uterine cavity normalization. This integrative treatment aligns with the trend toward uterus-preserving interventions and was shown to outperform more invasive options in several key domains.

Our findings support the hypothesis that combining TFA with HSC does not increase intra- or postoperative risks.^21^ The overall complication rates were low and similar between group 1 (8%) and group 2 (11%). These results are consistent with published data reporting low adverse event rates for both standalone TFA and hysteroscopic surgery.^3^;^20^ No severe or life-threatening complications were observed in either group. The nature of complications, such as bleeding, endometritis, and fibroid expulsion, was consistent with known risks associated with these procedures. Notably, intraoperative bleeding requiring the use of Foley catheter balloon tamponade in group 2 occurred during fibroid resection, with all cases involving fibroids larger than 3 cm, whereas in group 1, it occurred during endometrium resection. There were no differences in bleeding rates between group 1 and group 2. However, when the comparison was limited to the fibroid subgroups (group 1, 80 fibroid resections; group 2, 43 fibroid resections), a difference was recorded (*P* = 0.001). This suggests a lower risk of intraoperative bleeding in group 1, which may be attributed to prior fibroid ablation using TFA before operative HSC. Based on the authors’ experience, performing TFA before hysteroscopic fibroid resection (in cases with submucosal and intramural fibroids) substantially reduces intraoperative bleeding due to coagulation of vessels within the fibroid. Importantly, the addition of TFA did not increase the risk of severe intraoperative events or postoperative morbidity, even though it involved treatment of a broader spectrum of fibroid types (including intramural and transmural fibroids not accessible by HSC alone). The integration of real-time intrauterine ultrasound guidance in TFA likely contributed to the safe delineation of ablation zones and avoidance of thermal injury to surrounding tissues.

A key strength of the combined approach lies in its procedural complementarity. While operative HSC is limited to treating submucosal fibroids (FIGO types 0–2), TFA also enables treatment of intra- and transmural fibroids (FIGO types 3–6) in a safe, targeted manner, expanding the scope of minimally-invasive management without the need for laparotomy or laparoscopy, thus enabling broader eligibility for organ-preserving treatment.^3^ In our study, the patients in group 1 had a broader distribution of fibroid types, and mean fibroid size was larger than in the control group. Notably, despite these differences, the complication rates did not increase, and the symptom relief rate remained high. Furthermore, mean ablation time of under 9 minutes highlights the procedural efficiency of TFA, and its combination with HSC in a single surgical session minimizes anesthesia exposure and optimizes patient recovery.

The data also highlight procedural versatility of the combined approach. Nearly all fibroid types—excluding FIGO type 7—and a range of intrauterine pathologies can be managed in a single session by combining TFA with operative hysteroscopy, including polypectomy, endometrial ablation, and septum dissection. If septum dissection using monopolar energy is performed following TFA, the TFA electrodes must be removed beforehand. Moreover, the ability to address both submucosal and intramural fibroids in a single session offers procedural and economic advantages, and may reduce the need for future reintervention. An important consideration is a lack of routine histologic examination when performing TFA alone. This limitation is particularly relevant for women of advanced reproductive age or those with risk factors for endometrial pathology, in whom an undiagnosed endometrial abnormality could have significant clinical implications. However, a notable advantage of the combined approach used in our study is the possibility of obtaining direct visualization of the endometrial cavity via HSC, allowing for targeted biopsies or resection of suspicious areas when indicated. The integration of TFA and HSC enhances diagnostic safety by facilitating tissue sampling in selected cases, thereby addressing one of the key limitations of TFA as a standalone technique. We recommend that, in clinical practice, patients at an increased risk of endometrial pathology should undergo appropriate preoperative evaluation, including targeted endometrial biopsy, if necessary, to ensure optimal patient selection for TFA. Additionally, when using a combined approach, fibroid tissue obtained during hysteroscopic resection can be submitted for histologic examination.

Among the patients with available follow-up data in group 1, 83.3% reported improvement in AUB symptoms, with the highest response rate observed in those undergoing combined TFA and myomectomy (84%). These findings align with prior reports showing that TFA leads to sustained reductions in menstrual bleeding and improvements in health-related QoL over periods of up to 3 years.^6^;^7^

Laparoscopic (or robotic) myomectomy is the current standard for women desiring uterine preservation, especially when fertility is a priority, and typically results in substantial symptom improvement. However, a combination of TFA and HSC offers several comparative advantages. Because TFA is incisionless and performed transcervically under ultrasound guidance, there are no abdominal wounds and the recovery is faster. In the CHOICES (Comparing Hospital Outcomes and Impacts of Cervical Entry Surgery) trial,^25^ TFA had a markedly shorter operative time (90 vs 143 min) and dramatically shorter hospital stay (ca. 5 vs ca. 46 h), as compared with myomectomy. All perioperative costs (procedure, anesthesia, inpatient stay, pharmacotherapy, etc.) were considerably lower with TFA. The INSPIRE (Initial Comparison of Payer Costs for Sonata Relative to Standard of Care) study^26^ also found the total 12-month payer cost (including any follow-up care) of TFA to be less than half that of myomectomy. The avoidance of uterine incisions also translates to reduced postoperative pain and eliminates the risk of surgical site adhesions and uterine scar defects. This is particularly relevant for future pregnancy, as a transmural myomectomy scar can carry a small risk of uterine rupture in pregnancy and often necessitates cesarean delivery. In contrast, TFA involves ablation of fibroid tissue in situ, without weakening the uterine wall integrity. Early magnetic resonance imaging studies show that myometrial architecture remains intact after TFA.^27^ Furthermore, unlike surgical excision, TFA does not require suturing, potentially reducing intrauterine adhesion formation. Notably, a dedicated trial (the OPEN trial),^28^ where hysteroscopies were performed 6 weeks post-TFA, found no de novo intrauterine adhesions in 34 evaluated patients, including cases with fibroids abutting each other in the cavity. This suggests that TFA—even when when performed for the treatment of submucous / transmural fibroids—is unlikely to induce synechiae.

Data on fertility after TFA, while still accruing, are encouraging.^29^;^30^ A recent comprehensive case series compiled 89 post-TFA pregnancies in 72 women across multiple countries.^30^ These resulted in 55 known live births (19 vaginal and 35 cesarean deliveries). Importantly, there were no instances of uterine rupture, uterine dehiscence, or stillbirth in these pregnancies. The mean birthweight (ca. 3.28 kg) was within the normal range, and no increase in placenta accreta spectrum was observed. The overall first-trimester miscarriage rate was 22.8%, which is in line with expected rates for the general obstetric population in the age range. After excluding 2 patients with a known recurrent miscarriage history, the miscarriage rate was only approximately 10%.^30^ These outcomes suggest that pregnancies after TFA can be carried successfully without uterine scarring that follows myomectomy. In the combined approach, using HSC to resect submucous fibroids or polyps should theoretically improve fertility (by clearing the cavity), while TFA addresses intramural fibroids without adding scar tissue. Indeed, hysteroscopic treatment of intracavitary lesions is known to enhance fertility in appropriate candidates (eg, resecting a fibroid that impinges on the cavity). Thus, the combination of TFA and HSC may be especially suitable for women with both intramural and submucosal fibroids contributing to infertility or pregnancy loss. Although fertility outcomes following combined TFA and operative HSC are highly relevant from a clinical perspective, our retrospective analysis did not collect data regarding patient fertility status or post-treatment fertility outcomes. Future prospective studies specifically designed to assess fertility outcomes following this combined treatment approach are warranted to provide clinicians with clearer guidance when counseling women who plan pregnancy in the future.

Our study has several limitations. Despite promising findings, the retrospective design inherently limits control over potential confounding factors, and the absence of randomization may have introduced selection bias between the study groups. Furthermore, the control group was retrospectively selected from hospital records rather than through a predefined prospective protocol, potentially affecting intergroup comparability.

Although the post-hoc power analysis indicated an acceptable power of 87.2%, the overall sample size may still be insufficient to reliably detect rare complications with statistical certainty, limiting definitive conclusions regarding safety. The follow-up data were incomplete, with only 60% of the patients in the combined treatment group having available data, which may raise concerns about potential bias and the representativeness of our results. Additionally, no systematic follow-up or symptom outcome data were collected for the control group, precluding a robust direct comparison of long-term clinical effectiveness between the 2 approaches. Moreover, this study did not evaluate fertility outcomes. Although current literature indicates that transcervical radiofrequency ablation is not contraindicated for patients desiring future fertility,^3^;^4^ the absence of specific fertility data in our study limits the possibility of drawing definitive conclusions on reproductive outcomes. Given these considerations, our findings should be interpreted as preliminary and hypothesis-generating. Future prospective randomized studies with rigorous methodology, comprehensive long-term follow-up, and specific fertility end points are required to validate these results and fully define the clinical efficacy and safety of combining TFA with operative HSC.

## CONCLUSIONS

This retrospective analysis demonstrated that the combined use of TFA and operative HSC was safe and effective, with low complication rates similar to those observed in the patients undergoing operative HSC alone. The combined approach significantly reduced the risk of intraoperative bleeding during hysteroscopic myomectomy and achieved substantial symptom improvement in AUB. However, further prospective studies, particularly addressing long-term outcomes and fertility, are necessary to confirm these findings and establish definitive clinical guidelines.
